# New Insights into the *In Vitro* Antioxidant Routes and Osteogenic Properties of Sr/Zn Phytate Compounds

**DOI:** 10.3390/pharmaceutics15020339

**Published:** 2023-01-19

**Authors:** Gerardo Asensio, Marcela Martín-del-Campo, Rosa Ana Ramírez, Luis Rojo, Blanca Vázquez-Lasa

**Affiliations:** 1 Instituto de Ciencia y Tecnología de Polímeros (ICTP), CSIC. Calle Juan de la Cierva, 3, 28006 Madrid, Spain; 2 Facultad de Estomatología, Universidad Autónoma San Luis Potosí (UASLP), San Luis Potosí 78290, Mexico; 3 Centro de Investigación Biomédica en Red de Bioingeniería, Biomateriales y Nanomedicina, Instituto de Salud Carlos III, 28006 Madrid, Spain; 4 Interdisciplinary Platform for Sustainable Plastics towards a Circular Economy-Spanish National Research Council (SusPlast-CSIC), 28006 Madrid, Spain

**Keywords:** tissue engineering, bone regeneration, antioxidant, phytic acid, strontium, zinc

## Abstract

Sr/Zn phytate compounds have been shown interest in biomaterial science, specifically in dental implantology, due to their antimicrobial effects against *Streptococcus mutans* and their capacity to form bioactive coatings. Phytic acid is a natural chelating compound that shows antioxidant and osteogenic properties that can play an important role in bone remodelling processes affected by oxidative stress environments, such as those produced during infections. The application of non-protein cell-signalling molecules that regulate both bone and ROS homeostasis is a promising strategy for the regeneration of bone tissues affected by oxidative stress processes. In this context, phytic acid (PA) emerged as an excellent option since its antioxidant and osteogenic properties can play an important role in bone remodelling processes. In this study, we explored the antioxidant and osteogenic properties of two metallic PA complexes bearing bioactive cations, i.e., Sr^2+^ (SrPhy) and Zn^2+^ (ZnPhy), highlighting the effect of the divalent cations anchored to phytate moieties and their capability to modulate the PA properties. The *in vitro* features of the complexes were analyzed and compared with those of their precursor PA. The ferrozine/FeCl_2_ method indicated that SrPhy exhibited a more remarkable ferrous ion affinity than ZnPhy, while the antioxidant activity demonstrated by a DPPH assay showed that only ZnPhy reduced the content of free radicals. Likewise, the antioxidant potential was assessed with RAW264.7 cell cultures. An ROS assay indicated again that ZnPhy was the only one to reduce the ROS content (20%), whereas all phytate compounds inhibited lipid peroxidation following the decreasing order of PA > SrPhy > ZnPhy. The *in vitro* evaluation of the phytate’s osteogenic ability was performed using hMSC cells. The results showed tailored properties related to the cation bound in each complex. ZnPhy overexpressed ALP activity at 3 and 14 days, and SrPhy significantly increased calcium deposition after 21 days. This study demonstrated that Sr/Zn phytates maintained the antioxidant and osteogenic properties of PA and can be used in bone regenerative therapies involving oxidative environments, such as infected implant coatings and periodontal tissues.

## 1. Introduction

In recent years, researchers reported the importance of oxidative processes in the onset and pathogenesis of several human pathologies [1]. In particular, the latest evidence demonstrated the key role played by reactive oxygen species (ROS) in bone pathologies, especially those involving osteoimmunology, such as periodontitis [2,3,4,5]. An imbalance of in vivo ROS production has a direct impact on bone remodelling processes through the modulation of members of the tumour necrosis factor (TNF) superfamily [6]. Despite the fact that the exact mechanism remains unclear, it is known that the disruption of bone ROS homeostasis alters the levels of the signalling system formed by the receptor activator of nuclear factor kappa Β ligand (RANKL), the receptor activator of nuclear factor kappa-Β (RANK) and osteoprotegerin (OPG) [6,7]. Bone conditions, such as rheumatoid arthritis, osteoporosis and periodontitis, are mainly characterized by a detriment of bone-forming processes carried out by osteoblast cells in favour of bone resorption performed by osteoclasts. It is believed that osteocytes and osteoblasts upregulate RANKL expression, which promotes the differentiation of osteoclast precursors into osteoclast cells through direct binding to the RANK receptor when OPG does not act as a decoy receptor [8]. Therefore, large exposures to oxidative conditions foster osteoclastogenesis processes, leading to bone resorption and the release of proinflammatory cytokines [7,9]. Furthermore, hydroxyl radicals can worsen oxidative damage via the oxidation of polyunsaturated fatty acids in the lipid peroxidation process [10]. Lipid peroxides generated in lipid peroxidation have a longer lifetime than hydroxyl radicals and can diffuse longer distances, which gives them a greater harmful potential [11,12]. The toxic effects of lipid peroxides result in cellular membrane damage, the development of ferroptosis, the crosslinking of DNA and proteins and the further generation of ROS species [13].

Myo-inositol hexaphosphoric acid, or phytic acid (PA), is an abundant natural plant component with high biological potential. This compound is a predominant source of phosphate and Myo-inositol species in cereals and legumes and has been used as an organic source of P in fish feed and as an effective surface treatment agent against metals [14,15]. The characteristic chemical structure of PA, which is formed by 12 replaceable protons from 6 phosphate groups, enables a strong interaction with multivalent cations and proteins [16,17]. In this sense, PA was well documented as a powerful inhibitor of lipid peroxidation through the formation of ferrous complexes since the presence of free iron ions in the cellular environment catalyzes the formation of hydroxyl radicals in the Fenton reaction [18,19,20,21]. Furthermore, a growing interest is arising for further evaluation and understanding of the *in vitro* osteogenic processes in which PA is involved [22,23,24,25,26,27]. Some authors evidenced the key role of PA in the modulation of alkaline phosphatase (ALP) activity and tissue mineralization, the reduction of osteoclastogenesis processes via RANKL signalling, the differentiation of stem cells into an osteoblast phenotype and the regulation of the mRNA levels of bone biomarkers [22,23,24,26]. It is important to point out that PA intake was related to an increase in bone mineral density (BMD) [28] and a small loss of BMD when applied to a postmenopausal osteoporotic rat model [29]. Therefore, PA is presented as a potential compound for the treatment of ROS-mediated bone remodelling diseases, although a deep *in vitro* analysis is still needed to assess its osteogenic biological performance with pre-osteoblastic cell lineages.

In addition, PA ion exchange affinity can be employed to selectively modify its chemical structure by incorporating bioactive cations to expand the intrinsic biological properties of PA. Strontium and zinc cations are presented as two bioactive agents that are implicated in essential metabolic processes related to bone formation [30,31,32,33]. Sr^2+^ was extensively reported as a preferential promoter of osteoblast growth while inhibiting osteoclasts’ function, both *in vitro* and *in vivo* [32]. This cation exhibited a superior capacity in comparison to Ca^2+^ for the recalcification of low mineral zones affected by specific osteogenic bone diseases, exerting a superior transfer of Sr^2+^ to the areas with active bone remodelling [34]. Moreover, the beneficial *in vitro* and *in vivo* outcomes demonstrated for well-known strontium complexes, such as strontium ranelate and strontium folate, were not reproduced with the homologous Ca-based complexes, denoting the special role of Sr^2+^ in the promotion of bone-forming processes. [35,36]. Zn^2+^ is necessary for the correct development of the musculoskeletal system and was suggested to increase ALP activity and collagen synthesis *in vitro* with osteoblastic MC3T3-E1 [33,37,38]. In this context, our research group bonded Sr^2+^ and Zn^2+^ to a vitamin B9 ligand (folic acid) with the aim to provide new non-protein bioactive bone promoter compounds for their use in musculoskeletal regeneration based on the synergic action of the cations bound to folic acid moieties, modulating some osteogenic biomarkers *in vitro* with hMSCs cultures like ALP activity, matrix mineralization and the mRNA levels of some osteogenic-related genes [39,40]. In different studies, when they were incorporated into biohybrid scaffolds, they promoted the *in vivo* regeneration of tissues in critical-sized defects of rat calvarial bone defects and rabbit condyle [41,42].

In this sense, we recently developed two metallic phytate derivatives bearing Sr^2+^ or Zn^2+^ (named as SrPhy and ZnPhy) and studied their structural characteristics and antimicrobial activity, finding a synergistic effect of the cations with PA in the reduction of bacterial biofilm of *Streptococcus mutans* [43]. Here, we report the biological properties of the two metallic phytates that were found while exploring the complementary dual action of the cations and phytate rings on the PA properties. These phytate derivative antioxidants could be applied in bone tissue engineering and enhance the performance of other dental biomaterials and implants used in bone regeneration. Specifically, this study investigated the abilities of Sr/Zn PA derivatives regarding (i) their ferrous ions chelating activity and antioxidant properties using ferrozine/FeCl_2_ and a DPPH assay, respectively; (ii) the *in vitro* determination of their antioxidant behaviour via radical scavenging activity and the inhibition of lipid peroxidation using RAW264.7 cultures; (iii) the *in vitro* assessment of their cytotoxicity on hMSCs and RAW264.7 cells; and (iv) the evaluation of their capability for stimulating the differentiation of hMSCs into an osteoblastic phenotype.

## 2. Materials and Methods

### 2.1. Synthesis of Metallic Phytate Derivatives

Phytic acid sodium salt hydrate (C_6_H_18_O_24_P_6_·xNa.yH_2_O, from rice, also abbreviated as PA) was purchased from Sigma-Aldrich, strontium chloride hexahydrate (SrCl_2_·6H_2_O) was purchased from Acros Organics and zinc chloride (ZnCl_2_) was purchased from Fluka. All were used as received without further purification.

The Sr_4_C_6_H_12_O_24_P_6_·5H_2_O and Zn_6_C_6_H_12_O_24_P_6_·4H_2_O derivatives (named SrPhy and ZnPhy, respectively) were synthesized via a hydrothermal reaction of PA with the corresponding metal chloride salt and characterized following the same methodology described in a previous study [43]. Both complexes were obtained in high yields (>90%) and free of chloride impurities.

### 2.2. Radical Scavenger Activity Using a DPPH Assay

The radical scavenger activities (RSAs) of PA, SrPhy and ZnPhy were determined via the reduction in the absorbance of 2,2-diphenyl-1-picrylhydrazyl (DPPH, Merck KGaA, Darmstadt, Germany) ethanolic solution at different study times (15, 30, 60 and 120 min). Each phytate compound was solved in a mixture of ethanol:water (3:2) at 30 µg/mL. The DPPH reagent was also solved in ethanol:water (3:2) at a concentration of 0.127 mM. Vitamin E solution was prepared in the same conditions to assess the maximum RSA value and used as a positive control. Test samples (phytate solutions or vitamin E) and the DPPH solution were placed into a 96-well plate in a 1:1 proportion in dark conditions and left to react at room temperature. The absorbance of the mixture was measured spectrophotometrically at different time points at 515 nm in a Multi-Detection Microplate Reader Synergy HT (BioTek Instruments; Vermont, USA). The RSA was calculated using the following equation:

(1) $$\mathrm{RSA}\;\left(\%\right)=\left(\frac{A_{\mathrm{DPPH}}-A_{\mathrm{Sample}}}{A_{\mathrm{DPPH}}}\right)\cdot 100\;$$

where A_DPPH_ and A_Sample_ refer to the absorbance of DPPH and test sample solutions, respectively. The results were analyzed statistically using an ANOVA test at a significance level of *** *p* < 0.001 (Tukey’s means comparison). Data were expressed as mean value ± SD (*n* = 8).

### 2.3. Formation of Ferrous Chelate Complexes

The iron-chelating capacity of the phytate compounds was determined following the methodology described by Dinis et al. [44]. PA, SrPhy and ZnPhy were solved in the range between 2.5 and 30 µg/mL in deionized water (0.4 mL) and were mixed with 0.05 mL of 2 mM FeCl_2_ (Sigma Aldrich, St. Louis, MO, USA) solution. The mixture was left to react at room temperature for 15 min under dark conditions. Then, 0.2 mL of 5 mM ferrozine (Sigma Aldrich) solution was added, and the solution was made up to 4 mL total volume with ethanol. The ferrous chelate formation was measured spectrophotometrically by registering the absorbance of the solutions at 562 nm with a NanoDrop™ spectrophotometer. Ethylenediaminetetraacetic acid (EDTA; Sigma-Aldrich) solution (10 mM) and deionized water were used as the positive and negative controls, respectively. The chelating ferrous ions activity was calculated using the following equation:

(2) $$\mathrm{Ferrous}\;\mathrm{ions}\;\mathrm{chelating}\;\mathrm{activity}\;\left(\%\right)=100-\left(\frac{A_{\mathrm{Sample}}}{A_{H2O}}\;\cdot \;100\right)\;$$

where A_Sample_ and A_H2O_ refer to the absorbances of the phytate solutions and water blank, respectively. The assay was performed in triplicate for each sample and the results were given as the mean value ± standard deviation (SD). Statistical differences were assessed between samples at the same concentration using an ANOVA test (* *p* < 0.05) (Tukey’s means comparison).

### 2.4. In Vitro Biological Tests

#### 2.4.1. Cell Cultures

Cytotoxicity, cell proliferation and antioxidant properties were evaluated *in vitro* using human mesenchymal stem cells (hMSCs, P10576 Innoprot, Bizkaia, Spain) and RAW264.7 cells (murine macrophage cell line, Sigma Aldrich, 91062702). The hMSCs were expanded in Mesenchymal Stem Cell Medium Kit (MSCM, Innoprot, P60115). Afterwards, the cells were cultured with basal medium Dulbecco’s Modified Eagle’s Medium (basal-DMEM)-low glucose enriched with 110 mg/L of sodium bicarbonate and supplemented with 20% *v*:*v* of fetal bovine serum (FBS, Gibco, Waltham, MA, USA), 200 mM L-glutamine, 100 units/mL penicillin and 100 mg/mL streptomycin. Dulbecco’s Modified Eagles Medium-high glucose (DMEM-MHG, Sigma Aldrich, D6546), supplemented with 10% FBS, 2% L-glutamine and 1% penicillin/streptomycin, was used as the RAW264.7 culture medium.

Osteogenic properties were evaluated using the hMSCs. The cells were first expanded in MSCM for 24 h; then, the MSCM was replaced with the osteogenic medium DMEM Medium-low glucose (osteogenic-DMEM-LG, D5546 Sigma-Aldrich) enriched with 110 mg/L of sodium bicarbonate and supplemented with 20% *v:v* of fetal bovine serum (FBS, Gibco), 200 mM L-glutamine, 100 units/mL penicillin, 100 mg/mL streptomycin, 50 µg/mL ascorbic acid, 100 nM dexamethasone and 10 µM L-glycerol-2-phosphate; for the test samples, PA, SrPhy and ZnPhy were included at selected concentrations. Osteogenic-DMEM-LG culture medium without phytate compounds was used as a positive control.

All cell culture media were refreshed every 48 h and were maintained in a series 800 DH incubator (Thermo Scientific) at 37 °C in humidified air with 5% *v*:*v* CO_2_.

#### 2.4.2. Cytotoxicity

The cytotoxicities of the PA, SrPhy and ZnPhy compounds were studied using an AlamarBlue^®^ (AB^®^) assay with hMSCs and RAW264.7 cells. Due to the low solubility of phytate complexes, test samples were solved in a culture medium:PBS mixture (1:1) at a final concentration of 0.03 mg/mL, and basal-DMEM:PBS mixture (1:1) or DMEM-MHG:PBS (1:1) was employed as a positive control for each cell lineage, respectively. Several dilutions of the corresponding tested compound were prepared (2.5, 5, 10, 20, 30 µg/mL) by employing the corresponding culture medium:PBS mixture (1:1). Both cell lineages were seeded in 96-well plates at a density of 1×10^4^ cells/well. The culture medium was removed and cells were treated with the dilutions of the tested compounds for 24 h. Then, the medium was replaced by an AB^®^ (Invitrogen) solution (10%, *v:v*) prepared in DMEM-LG phenol red-free, and the plates were incubated at 37 °C for 3 h. The medium was collected and the fluorescence was measured at 590 nm after excitation at 560 nm using a microplate reader (Biotek Synergy HT spectrophotometer). The positive control was used as the calculation of 100% cell viability. The results of the tested samples were statistically evaluated relative to the positive control (* *p* < 0.05, ** *p* < 0.005 and *** *p* < 0.001) and SrPhy and ZnPhy samples relative to PA (# *p* < 0.05, ## *p* < 0.005 and ### *p* < 0.001) using the one-way ANOVA Tukey’s means comparison. Data were given as the mean value ± SD (*n* = 8).

#### 2.4.3. Antioxidant Activity Using Reactive Oxygen Species Quantification

ROS production was assessed by employing the fluorescence probe 2,7′-dichlorodihydrofluorescein diacetate (DCFH-DA). Compounds were solved in a mixture of DMEM-MHG:PBS at a final concentration of 0.03 mg/mL. RAW264.7 cells were seeded at a density of 2.5 × 10^4^ cells/well in 96-well plates and cultured overnight. The culture medium was substituted by the corresponding phytate solution or a mixture of culture medium:PBS (1:1), and the cells were incubated for 24 h. Then, a DCFH-DA probe was added to each well at a final concentration of 40 µM and incubated for 45 min under dark conditions. The probe was removed, and the cells were washed twice with PBS. Macrophage activation was carried out via stimulation with H_2_O_2_ (100 mM per well) for 30 min. Fluorescence was measured with a Multi-Detection Microplate Reader with an excitation/emission of 485/528 nm and imaging was acquired with a Nikon TE2000-S Eclipse Microscope. To compare the results, cells treated with culture medium:PBS (1:1) and stimulated with H_2_O_2_ were taken as the positive control. Basal levels of ROS were determined using macrophage samples incubated with culture medium:PBS (1:1) and no activation treatment (negative control). Data were normalized to the positive control, which was estimated as 100% of ROS production. Results obtained were evaluated using the one-way ANOVA test (Tukey’s means comparison) at a significance level *** *p* < 0.001 and were expressed as the mean value ± SD (*n* = 8).

#### 2.4.4. Qualitative and Quantitative Assessment of Lipid Peroxidation

Lipid peroxidation was evaluated in RAW264.7 cells by employing a qualitative Lipid Peroxidation Assay Kit (cell-based, Abcam 243377), and quantitatively using an MDA Assay Kit (competitive ELISA, Abcam 238537) following the manufacturer’s instructions. All compounds were solved at 0.03 mg/mL in a DMEM-MHG:PBS mixture (1:1). For both tests, the RAW264.7 cells were seeded at a concentration of 10^6^ cells/well in 6-well plates and incubated for 24 h with the corresponding test sample solutions or with a mixture of culture medium:PBS (1:1) for the controls. Macrophage activation was carried out using the addition of Fe_2_SO_4_·7H_2_O at a concentration of 50  μM/well and H_2_O_2_ at 50 µM/well, with incubation for 4 h. The negative control was taken to be cells treated with culture medium:PBS (1:1) and without lipid peroxidation induction. The positive control was the cells treated with the H_2_O_2_/Fe_2_SO_4_·7H_2_O system but without phytate compounds. The qualitative determination of MDA production was evaluated via fluorescence microscopy images using a Nikon TE2000-S Eclipse Microscope. For the ELISA kit, the percentage of lipid peroxidation inhibition was calculated regarding the positive control (taken as 100%). The data obtained were normalized according to the DNA content determined with a PicoGreen dSDNA quantitation kit (Molecular Probes) following the manufacturer’s instructions and analyzed for statistical significance using the one-way ANOVA test through Tukey’s means comparison (* *p* < 0.05, ** *p* < 0.005 and *** *p* < 0.001). The experiments were performed in duplicate for each sample and the results were given as the mean value ± SD (*n* = 2).

#### 2.4.5. Cell Viability

Cell viability was evaluated in the hMSCs with an AB^®^ assay at 3, 7, 14 and 21 days. The cells were seeded at a density of 2 × 10^4^ cells/well in 24-well plates and cultured with MSCM for 24 h; afterwards, the medium was replaced by solutions of the tested compounds in basal-DMEM at selected concentrations. PA and ZnPhy were solved at 5 and 10 µg/mL (*n* = 8) in basal-DMEM, but due to the low solubility of SrPhy, it was first solved in PBS at 20 µg/mL and diluted with a mixture of basal-DMEM:PBS (1:1) to the desired concentration. Cells that were not treated with phytate compounds were used as the positive control. After each study time, the medium was replaced by an AB^®^ (Invitrogen) solution (10%, *v:v*) in DMEM-LG phenol red-free and incubated at 37 °C for 3 h. The medium was collected and fluorescence was measured at 560 nm after excitation at 590 nm using a microplate reader (Biotek Synergy HT spectrophotometer). Cell viability (%) was calculated relative to the negative control, which was considered the maximum cell viability (100%). The data obtained were statistically compared using the one-way ANOVA (Tukey’s means comparison) relative to the negative control (* *p* < 0.05, ** *p* < 0.005 and *** *p* < 0.001) and PA (# *p* < 0.05, ## *p* < 0.005 and ### *p* < 0.001). The results were expressed as the mean value ± SD (*n* = 8).

#### 2.4.6. Alkaline Phosphatase Activity

Quantification of the alkaline phosphatase (ALP) activity was determined by monitoring the increase in absorbance produced by the reaction between *p*-nitrophenyl and the ALP found in samples in which *p*-nitrophenol (coloured) was formed. The hMSCs were seeded at a concentration of 6 × 10^4^ cells/well in 6-well plates and cultured with MSCM for 24 h. Then, the MSCM was replaced by osteogenic-DMEN-LG with phytate compounds (5 and 10 µg/mL) or only by an osteogenic-DMEM culture medium. The selected time points were 3, 7 and 14 days. Cell detachment was carried out by washing with PBS twice, followed by resuspension with cold distilled water. Three cycles of thermal shocks from liquid nitrogen to a warm water bath (37 °C) were performed for cellular lysis. For the quantification of ALP, 20 µL of each sample were placed on 96-well plates and incubated with 60 µL of a 0.2% *w*:*v* *p*-nitrophenyl (Sigma Aldrich) solution in 1 M diethanolamine for 45 min at 37 °C under dark conditions. The reaction was stopped via the addition of 80 µL of 0.2 mM EDTA solution and absorbance was measured at 405 nm in a microplate reader (Biotek Synergy HT spectrophotometer). The results were normalized to DNA by employing the PicoGreen dSDNA quantitation kit (Molecular Probes) following the manufacturer’s instructions. The experiments were performed in triplicate for each sample and the data obtained were expressed as mean value ± SD. The data were statistically analyzed using the one-way ANOVA (Tukey’s means comparison) relative to the positive control (* *p* < 0.05, ** *p* < 0.005 and *** *p* < 0.001) and PA (# *p* < 0.05, ## *p* < 0.005 and ### *p* < 0.001). The results were expressed as the mean value ± SD (*n* = 3).

#### 2.4.7. Qualitative and Quantitative Determination of Matrix Mineralization Degree

The production of a calcium matrix induced by the exposure to phytate compounds was evaluated via specific staining with an alizarin red (AzR) reagent (Sigma Aldrich) after 21 days of culture. The hMSCs were seeded at a density of 6 × 10^4^ cells/well in 6-well plates for 24 h and then incubated with the corresponding compound solution (5 and 10 µg/mL) or osteogenic-DMEM-LG for the positive control. At each study time, the wells were washed once with PBS and fixed with cold ethanol at 70% *v:v* for 1 h at 4 °C. Then, wells were washed once with distilled water and stained with 1 mL of a 40 mM AzR solution for 30 min at room temperature. Unspecific staining was removed via washing with distilled water (5 times). Microscopic images were taken with a Nikon TE2000-S Eclipse Microscope. For the quantification of calcium deposits, a 10% *w:v* cetylpyridinium chloride (CPC, Sigma Aldrich) solution solved in 10 mM disodium phosphate buffer was employed. Then, 1 mL of CPC was added to each well and left to react at room temperature for 30 min. Afterwards, aliquots were taken and the absorbance was measured at 550 nm. A calibration curve was constructed using serial dilutions of AzR in CPC solution (from 0.75 to 4 mM of AzR, R^2^ = 0.999). A one-way ANOVA via Tukey’s means comparison was performed at * *p* < 0.05, ** *p* < 0.005 and *** *p* < 0.001 to assess the statistical differences between the phytate compounds and the positive control. The data were expressed as the mean value ± SD (*n* = 3).

## 3. Results and Discussion

The development of new bioactive molecules for the regeneration of bone tissues that overcomes the limitations of currently used growth factors is highly demanded. The production process of typical protein cell-signalling factors (i.e., bone morphogenetic proteins, insulin growth factors) is complex, leads to high production costs, and their low stability hinders their handling and storage. Furthermore, several side effects were found for biologically active concentrations [45]. Thus, the increased stability and accessibility of bioactive cations, such as Sr^2+^ and Zn^2+^, make them potential candidates for bone regeneration. In fact, strontium ranelate was administered to treat osteoporotic diseases; however, its negative side effects, such as those related to a significant increase in non-fatal heart attacks and blood clotting [46,47], have drastically restricted its use and have motivated the search for new organic carriers of bioactive cations. In this sense, our group developed metallic complexes with organic moieties, such as phytic acid, that can increase their osteogenic effects [39,43]. Herein, the goal of the present study was to analyze the role of these phytate compounds regarding their antioxidant and osteogenic properties for their potential use in bone tissue engineering applications, especially when the regeneration of tissues is associated with oxidative stress conditions.

### 3.1. Antioxidant Capacity Assessed Using a DPPH Assay

**Figure 1:**
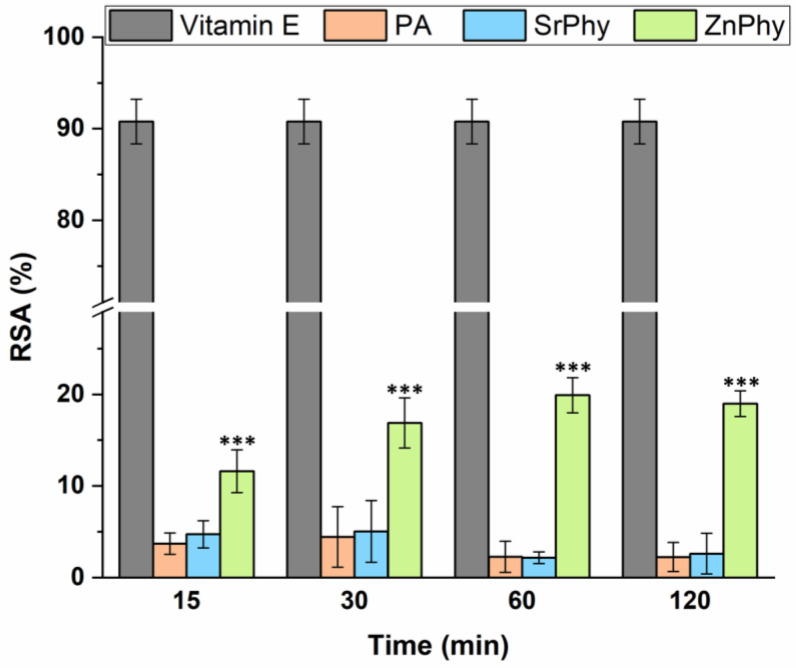
Radical scavenger activities of phytate compounds at 30 µg/mL that were obtained after 15, 30, 60 and 120 min of incubation with a DPPH solution. Vitamin E was taken as the positive control to assess the maximum RSA achieved. The results are represented as the mean RSA value (%) ± SD (*n* = 8), and were statistically evaluated using an ANOVA (*** *p* < 0.001, Tukey’s test).

Free radicals play an essential role in the regulation of cellular functions via redox signalling, but a sustained overproduction is related to the initiation of oxidative stress processes [48]. The RSA of phytate compounds was evaluated through the neutralization of DPPH free radicals and the results obtained are shown in Figure 1. All the compounds were tested over time at a concentration of 30 µg/mL. PA and SrPhy showed no radical scavenger activity at any study time. This was expected for PA since it was reported to inhibit iron-catalyzed hydroxyl radical formation via the Fenton-type reaction but it has no effect towards DPPH [18,19]. The same could be assumed for SrPhy since no references reported the role of the Sr^2+^ cation as a free radical scavenger. However, a decrease in free radicals was registered for ZnPhy with an increasing tendency over time. The RSA achieved a plateau after 1 h of reaction, reaching a maximum RSA value of 21%. The ZnPhy derivative was predicted to exhibit free radical scavenger activity due to the role of Zn^2+^ as a powerful antioxidant [37,49,50].

### 3.2. Ferrous Ions’ Chelating Ability

**Figure 2:**
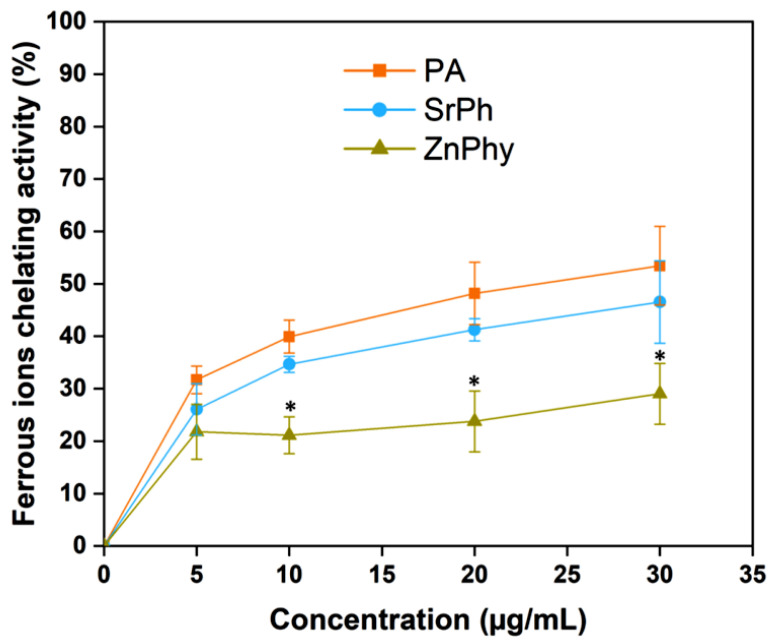
Ferrous complex formation capacities of PA, SrPhy and ZnPhy at different concentrations in the presence of ferrozine. Statistical differences between samples at each concentration were assessed (* *p* < 0.05) using an ANOVA test (Tukey’s means comparison). Data are represented as the mean value (%) ± SD. The experiment was conducted with triplicate samples.

Ferrous ions constitute one of the main factors for the onset and propagation of oxidative processes [51]. The presence of free iron ions in the cellular microenvironment is responsible for the production of hydroxyl radicals in the Fenton reaction, which causes oxidative damage in mammalian tissues [52]. Hence, iron-catalyzed oxidative reactions will be hampered in the presence of chelating compounds with ferrous ion affinity. PA antioxidant properties are well documented as functioning via this route [18], and thus, we used the ferrozine colorimetric method in the presence of FeCl_2_ to evaluate the capacity of phytate compounds to prevent the Fenton reaction. Figure 2 shows the tendency of chelating activity produced by PA, SrPhy and ZnPhy in the range where the phytate complexes were soluble. All compounds underwent a concentration-dependent trend in which the higher the concentration of the compound, the more chelating capacity was achieved following the order of PA > SrPhy > ZnPhy, reaching values of 52, 48 and 29%, respectively. In the case of SrPhy, no significant differences were observed relative to PA, indicating a similar ion-exchange ability between Sr^2+^ and Fe^2+^, and hence, a similar antioxidant power to PA via this pathway, while ZnPhy exhibited a lower chelating activity than PA and SrPhy across the concentration range, which was attributable to the lower stability constant in the solution for the PA complexes with Fe^2+^ than Zn^2+^ [53,54]; we found that both phytate derivatives presented antioxidant activity via the inhibition of iron-catalyzed oxidative reactions via the Fenton reaction, although it was lower for the ZnPhy complex. However, the impairment for the ZnPhy derivative was countered by its radical scavenger activity against DPPH radicals (Figure 1).

### 3.3. In Vitro Biological Performance

#### 3.3.1. Cytotoxicity

**Figure 3:**
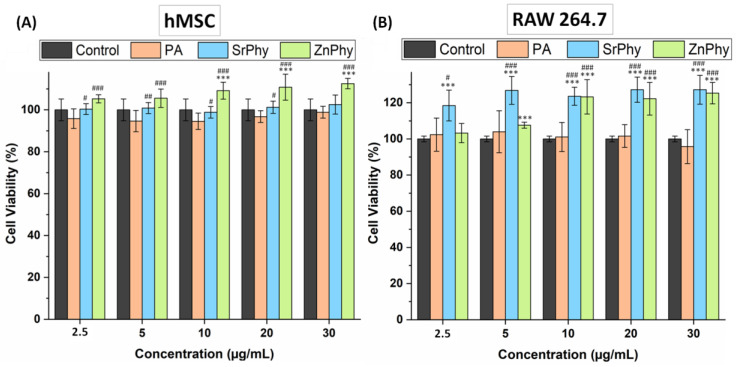
Cytotoxicity assessment of phytate compounds with (**A**) hMSC and (**B**) RAW264.7 cultures. An ANOVA was performed between PA, SrPhy and ZnPhy with control samples (basal-DMEM) (*** *p* < 0.001) and between SrPhy and ZnPhy relative to PA (# *p* < 0.05, ## *p* < 0.005, ### *p* < 0.001) at each concentration in both cases (Tukey’s means comparison). The results are displayed as the mean value (%) ± SD (*n* = 8).

The cytotoxicity of phytate compounds was studied with hMSCs and RAW264.7 cells using an AB^®^ assay in the concentration range from 2.5 to 30 µg/mL, in which the metal complexes were soluble in a culture medium. The results were normalized to the control group (basal-DMEM) cultured on plastic plates and expressed as a percentage of the cell viability (Figure 3). ZnPhy showed a remarkable concentration-dependent effect with both cell lineages, exhibiting an increased tendency regarding cell viability with increased concentration. In the case of the hMSCs, their viability was significantly increased (### *p* < 0.001) relative to PA over the whole range of concentrations assayed, and significant differences were also achieved at 10, 20 and 30 µg/mL relative to the control group (*** *p* < 0.001); meanwhile, the RAW264.7 cultures showed significantly higher cell viabilities in the presence of ZnPhy in the range of 5–30 µg/mL when compared with the control group (*** *p* < 0.001) and also at 10, 20, and 30 µg/mL relative to PA (### *p* < 0.001). In the case of SrPhy, a notable concentration-dependent effect was only observed for RAW264.7 cultures in which the cell viability was significantly increased (*** *p* < 0.001) at all tested concentrations in comparison with the control, and also reached significant differences relative to PA. When the hMSC cultures were used, the cell viability did not differ from the control samples, but did significantly increase relative to PA at 2.5, 5, 10 and 20 µg/mL (# *p* < 0.05 and ## *p* < 0.005, respectively). The overall results exhibited no cytotoxic effect on any tested compound and even enhanced the cell viability for both hMSC and RAW264.7 lineages when cultured in the presence of the synthetic phytate complexes.

#### 3.3.2. Quantification of ROS Production

**Figure 4:**
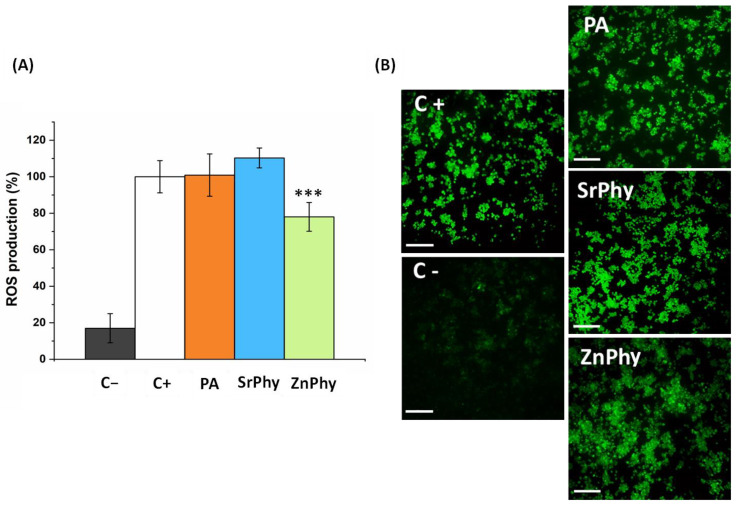
(**A**) Quantitative analysis of ROS production by RAW264.7 cultures. The results were expressed relative to the positive control, which was referred to as 100% ROS production. An ANOVA test was performed between samples at the significant level *** *p* < 0.001 (Tukey’s test). (**B**) Fluorescence microscopy images were obtained for positive and negative controls (left), and PA, SrPhy and ZnPhy samples (right). Scale bars correspond to 100 µm. The positive control refers to cells stimulated with H_2_O_2_ and the negative control corresponds to untreated cells. The results are presented as the mean ROS production (%) ± SD (*n* = 8).

Sustained oxidative stress originating from a prolonged ROS imbalance was linked with the promotion of macrophage differentiation into osteoclast cells, which fostered osteoclastogenesis processes and the pathogenesis of bone [55,56]. We monitored the ROS production of RAW264.7 cultures with the DCFH-DA probe since macrophages can regulate ROS production based on the signals received from the environment [7]. The presence of ROS produces intracellular deacetylation of the non-fluorescent DCFH-DA species to form 2,7′-dichlorodihydrofluorescein (DCFH), which is rapidly oxidized to the fluorescent compound dichlorofluorescein (DCF), and thus, the higher intensity of fluorescence detected can be related to an increase in the production of ROS in an *in vitro* model. Figure 4 presents the quantitative and qualitative assessments of the radical scavenger activity exhibited by phytate compounds. For the positive control (referred to as 100% of the ROS production), the cells were activated with H_2_O_2_. Only ZnPhy was able to reduce the ROS content, reaching a decrease of 20% in the intensity of the fluorescence signal (Figure 4A). The ROS production was further analyzed using fluorescence microscopy and the results are displayed in Figure 4B. Negative control images used to establish basal ROS levels are shown. The macrophage cultures under PA and SrPhy treatment did not show a decrease in the fluorescence intensity emitted by the probe, in contrast to ZnPhy samples in which a less bright signal was registered. Accordingly, the same tendency was observed in the antioxidant DPPH assay discussed above (Figure 1). These results demonstrated that the formation of a Zn^2+^ complex enabled the conferring of new antioxidant properties to PA as a free radical scavenger in addition to its native potential for inhibiting lipid peroxidation. Moreover, this finding was in agreement with several studies that reported this effect for zinc [57,58,59,60], zinc complexes with polymers [61] and zinc complexes with organic ligands [62,63], which showed increased RSA values and the maximum inhibition of ROS production with an increase in Zn^2+^ in the medium.

#### 3.3.3. Lipid Peroxidation Inhibition

**Figure 5:**
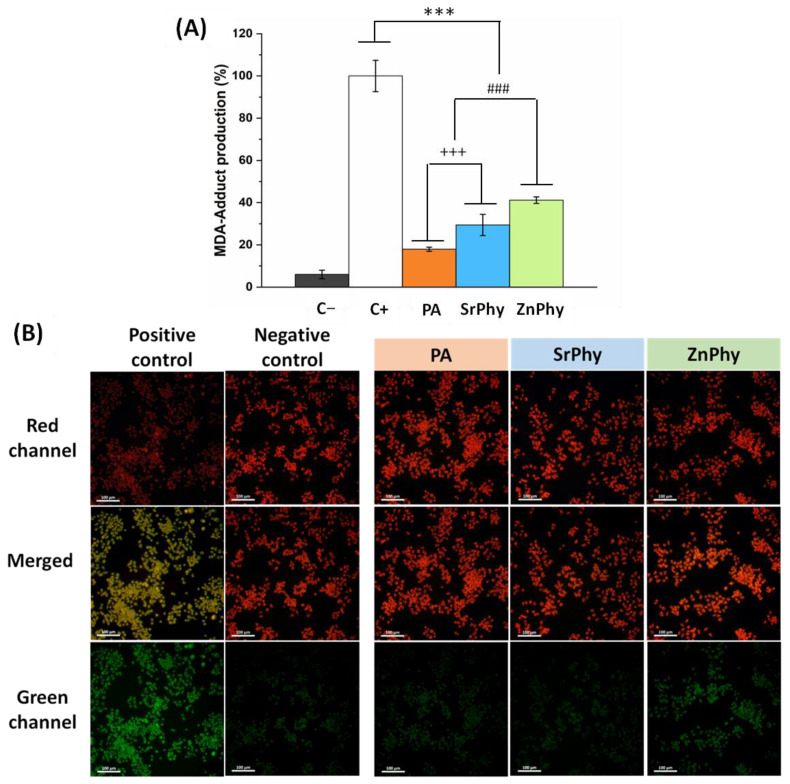
(**A**) Quantitative assessment of the MDA production as determined with an ELISA test with RAW264.7 cultures. The positive control corresponds to activated macrophages (Fe_2_SO_4_·7H_2_O/H_2_O_2_ treatment). Statistical differences were evaluated at the significance levels *** *p* < 0.001 relative to the positive control, ### *p* < 0.001 for ZnPhy relative to PA and SrPhy, and +++ *p* < 0.001 between PA and SrPhy (Tukey’s test). (**B**) Qualitative determination of lipid peroxidation using fluorescence imaging. Green fluorescence corresponds to activated cells (positive control) and the red signal was emitted by non-treated cells (negative control). Scale bars correspond to 100 µm. Results were presented as the mean value (%) ± SD (*n* = 2).

It is well known that iron induces lipid peroxidation in macrophage cultures [64]. Malondialdehyde (MDA) is one of the most common degradation products generated in the early stages of lipid peroxidation and was recently used as a biomarker for the measurement of lipid peroxidation [65]. The production of this adduct in stimulated RAW264.7 cultures can be quantitatively determined using an ELISA kit assay to avoid the limitations of other methods, such as the detection of thiobarbituric-acid-reacting substances, namely, the TBAR assay, which may lead to more imprecise results when applied to biological samples due to MDA–TBA adduct reactivity [66]. Figure 5A shows the results obtained in the ELISA test normalized against DNA content and expressed as a percentage of lipid peroxidation. Statistical analysis was performed relative to the positive control (100% lipid peroxidation), which referred to activated macrophages via Fe_2_SO_4_·7H_2_O/H_2_O_2_ without phytate supplementation. Under a 30 µg/mL dose, the macrophage cultures treated with phytate compounds were able to significantly reduce the amount of MDA adduct found in all experimental groups (*** *p* < 0.001), which was in accordance with the ferrous chelating capacity of each compound discussed above (Figure 2). The lowest value of MDA production was reached by PA (18%), followed by SrPhy (30%) and ZnPhy (41%). Significant differences were also found between ZnPhy relative to PA and SrPhy (### *p* < 0.001), and between SrPhy and PA (+++ *p* < 0.001). Lipid peroxidation was further qualitatively analyzed through a colourimetric method for the identification of non-affected (red channel) and activated macrophages (green) [67,68,69]. Pictures are given in Figure 5B. The positive and negative controls are shown to establish the colour range. The images of the cultures treated with PA and SrPhy revealed an intense red fluorescence and almost no green signal, which implied a high inhibition of lipid peroxidation. Similar behaviour was exhibited by the ZnPhy samples, but a greater green fluorescence was observed in the images. These results were in agreement with the quantitative data obtained in the ELISA test (Figure 3A) since ZnPhy was the less effective compound regarding the reduction of MDA levels.

In summary, our research suggested that the cation bonded to phytate anion conferred a complementary dual effect on the PA antioxidant properties, and hence, in lipid peroxidation inhibition. The results showed that the SrPhy derivative reduced lipid peroxidation to a higher extent relative to that of the Zn one, probably due to its ion-exchange ability with ferrous cations, as observed in Figure 2. Likewise, zinc intake was related to protective effects for the prevention of lipid peroxidation *in vivo* [70,71] acting as an antagonist of pro-oxidative transition metals, such as iron or copper, due to their replacement at characteristic binding sites and the further inhibition of the catalytic decomposition of hydrogen peroxide to hydroxyl radicals [37,72]. In accordance with this, ZnPhy samples reduced MDA-adduct formation relative to the control but exhibited the highest levels of MDA production relative to SrPhy and PA, which could be attributed to its lower ferrous complex formation ability (Figure 2); however, the zinc presence conferred free radical scavenger potential, as shown in the DPPH and ROS production in the RAW264.7 culture tests (Figure 1 and Figure 4).

#### 3.3.4. Cell Viability

**Figure 6:**
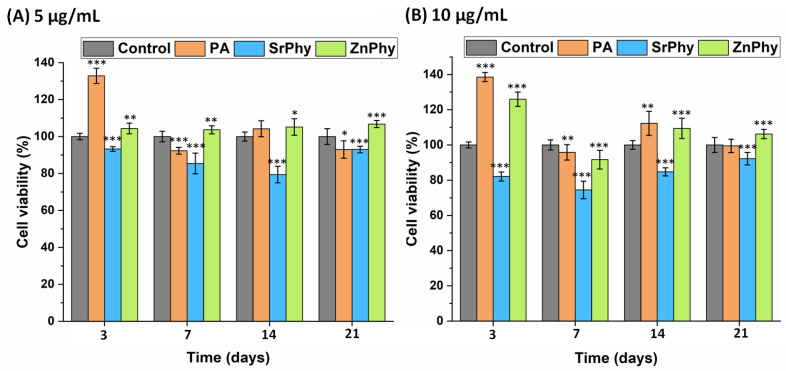
Metabolic activity exhibited by the hMSC cultures in the presence of PA, SrPhy and ZnPhy at (**A**) 5 µg/mL, and (**B**) 10 µg/mL. Cells were incubated over 3, 7, 14 and 21 days of culture. Means comparison was performed using an ANOVA analysis against the control group (basal-DMEM) for each time point (* *p* < 0.05, ** *p* < 0.005, *** *p* < 0.001; Tukey’s test). The results are expressed as the mean percentage value (%) ± SD (*n* = 8).

The metabolic activity of the hMSCs after 3, 7, 14 and 21 days of culture was determined using an AB^®^ assay. Two concentrations were evaluated for all compounds (5 and 10 µg/mL) based on previous reports that delimited the active biological range of PA in the micromolar region [73]. The results obtained were statistically analyzed relative to the control group (basal-DMEM) and PA (Figure 6). Phytate derivatives supported good cell proliferation of the hMSCs at all study time points for both concentrations tested. In particular, the SrPhy sample exhibited cell viabilities over 70% for both doses at all study times but the viability decreased for the first few time points. After 21 days of culture, cell viability recovered (>90%) but did not reach the control values. In contrast, ZnPhy promoted a significant increase in the proliferation rate of hMSCs at all times for both concentrations, except at 7 days with the 10 µg/mL dose, where a significant decrease in cell viability was achieved without reaching toxicity. The PA exhibited an intermediate time-dependent tendency. It reached the maximum viability under all conditions evaluated after 3 days of culture (### *p* < 0.01) and significantly decreased after 7 days. The metabolic activity recovered after 14 days, but slightly reduced its value again at 21 days under the 10 µg/mL dosage, and was significantly lower for 5 µg/mL (### *p* < 0.001).

These results, along with the improved cytotoxicity (Figure 3), confirmed the suitability of PA and SrPhy/ZnPhy derivatives for their application as biocompatible bone drugs and agreed with the reported literature. Phytate supplementation in the micromolar range, tested with hMSCs and MC3T3-E1 osteoblast cultures, showed a similar effect on the regulation of cell viability, which always surpassed 70% [24,26].

#### 3.3.5. ALP Activity Quantification

**Figure 7:**
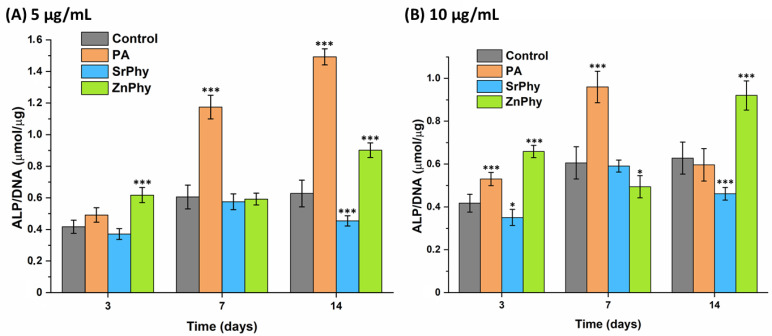
Quantitative assessment of ALP activity found in the hMSC cultures after 3, 7 and 14 days of treatment with PA, SrPhy and ZnPhy under a dose of (**A**) 5 µg/mL and (**B**) 10 µg/mL. The data were normalized against DNA content measured using a PicoGreen dSDNA quantitation kit. An ANOVA test was performed between the samples and the control group (osteogenic-DMEM) at each study time to establish the statistical differences at significant levels (* *p* < 0.05, *** *p* < 0.001; Tukey’s test). The results are expressed as the mean ALP/DNA (µmol/µg) ± SD (*n* = 3).

ALP enzyme activity is regularly used as a biomarker for the estimation of *in vitro* bone regeneration ability since it is expressed in the early stages of osteoblast differentiation. The hMSCs were cultured for 3, 7 and 14 days with osteogenic-DMEM medium supplemented with two doses (5 and 10 µg/mL) of each phytate compound. Osteogenic-DMEM medium alone was employed as the control group for the statistical differences, and the data were normalized against DNA content (Figure 7). The results showed that phytate compounds exerted a modulator role in ALP activity in a dose- and time-dependent manner. Remarkable results were obtained for ZnPhy, as it exhibited the greatest ALP expression at 3 and 14 days when the lowest dose (5 µg/mL) was tested (*** *p* < 0.001), and reached significant differences relative to the control group at 14 days of culture time at a dosage of 10 µg/mL (*** *p* < 0.001). Similarly, cells treated with PA at a concentration of 5 µg/mL significantly increased the ALP activity at 3 and 7 days (*** *p* < 0.001), and reached the highest value when a 10 µg/mL dose was applied at 7 and 14 days (*** *p* < 0.001). The dose effect on ALP activity seemed to increase its expression when a higher concentration of PA was applied. In the case of SrPhy, we observed the maintenance of ALP activity levels and even a significant downregulation at 3 days (* *p* < 0.05) and 14 days (*** *p* < 0.001) for a concentration of 5 µg/mL, and at 14 days for 10 µg/mL (*** *p* < 0.001).

Our findings corroborated the particular role of PA in the regulation of ALP activity and were consistent with previous reports. Mora-Boza et al. quantified the ALP activity with hMSCs cultured in basal conditions and registered an upregulation of this biomarker when a dose of 10 µg/mL was assayed [26]. These results are in agreement with Addison et al. since they also observed an increase in ALP activity when MC3T3-E1 cells were exposed to 4 µM PA in differentiation media, while tissue mineralization was inhibited [24]. Moreover, Arriero et al. found a different effect according to the used cell lineage, obtaining a remarkable increase in ALP activity when hUC-MSCs were tested, contrary to MC3T3-E1 cultures [23].

#### 3.3.6. Matrix Mineralization Degree

**Figure 8:**
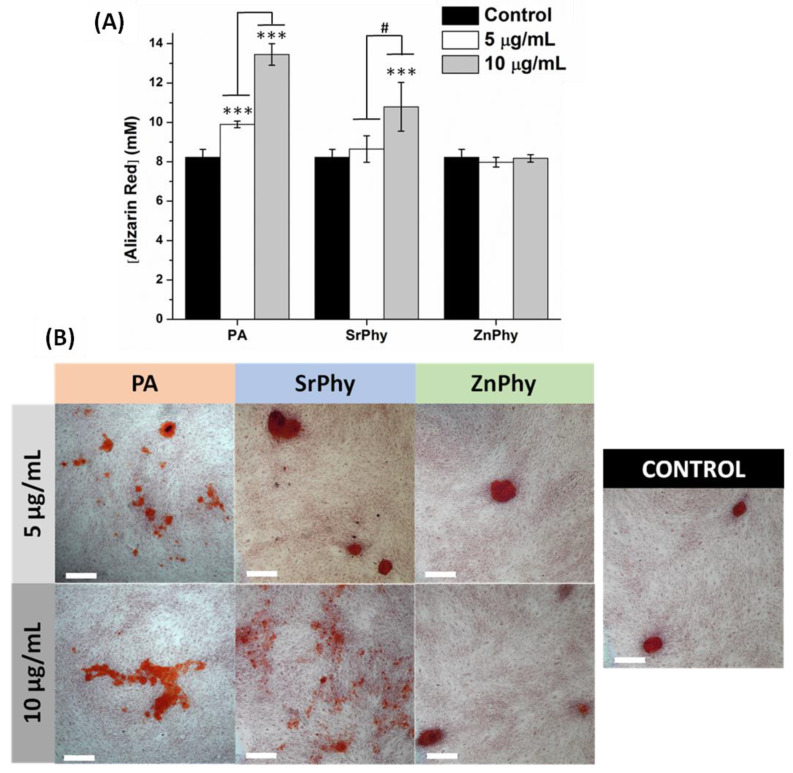
(**A**) Quantitative determination of extracellular matrix mineralization with hMSCs cultures in the presence of PA, SrPhy or ZnPhy at 5 and 10 µg/mL after 21 days. The results for each concentration were statistically compared relative to the control group (osteogenic-DMEM) (ANOVA, *** *p* < 0.001, Tukey’s test), and between the two concentrations tested in each compound (ANOVA, # *p* < 0.001, Tukey’s test). The results are represented as the mean AzR concentration (mM) ± SD (*n* = 3). (**B**) Representative microscopic images (4×) of the AzR staining after 21 days of culture. Scale bars correspond to 500 µm.

The ability of osteogenic drugs to induce tissue mineralization is a key feature of their potential application in bone tissue engineering. We studied the capacity of phytate compounds to enhance bone matrix formation at two concentrations (5 µg/mL and 10 µg/mL) after 21 days of culture (Figure 8) by using the alizarin red (AzR) dye, which is a common assay used for the identification of calcium deposits and the evaluation of tissue mineralization. Osteogenic-DMEM differentiation medium without any phytate compound was used as the positive control for the statistical analysis. The AzR quantification results are shown in Figure 8A. After 21 days of culture, the PA samples (at both 5 and 10 µg/mL) and SrPhy in the 10 µg/mL group were significantly higher than the control (*** *p* < 0.001). However, ZnPhy samples were able to produce the same calcification levels as the control group for both studied concentrations. Interestingly, when we analyzed the dose effect on calcium deposition exhibited by the same compound at each study time, a concentration-dependent performance was observed with a significantly increased mineralization ability as higher doses of SrPhy were tested (# *p* < 0.001). The AzR-stained culture images are displayed in Figure 8B. The results obtained were consistent with the quantitative data since the PA samples exhibited the highest mineralization degree in terms of the size and amount of calcium nodules. The SrPhy dose played a modulator effect on calcium deposition mode, allowing for the formation of big isolated nodules at 5 µg/mL, while more homogeneous staining with smaller nodules took place throughout the extracellular matrix when cells were exposed to a 10 µg/mL dose. Finally, the presence of ZnPhy led to the formation of similar nodules in size and quantity relative to control samples.

Only a few studies in the literature investigated the potential of PA to induce tissue mineralization when it is applied with osteoblastic- or preosteoblastic-like lineage cultures. Discrepancies regarding whether mineralization is favoured were discussed in terms of a correlation between the cell lineage and supplementation mode. An inhibitory effect of tissue mineralization was found with MC3T3-E1 and human umbilical cord mesenchymal stem cells despite the cells being properly differentiated into an osteoblast phenotype and osteopontin (inhibitor of osteoblast mineralization) [23,24]. In contrast, when PA was applied in numerous biomedical systems, all the studies reported increased biomineralization, regardless of the cell lineage [25,27,74,75,76,77]. Regarding SrPhy, numerous authors reported the ability of Sr^2+^ to overexpress the mRNA levels of osteocalcin, which is a gene involved in the mineralization of tissues, at 14 and 21 days [78,79,80]. These reported studies support our results displayed above and confirm the crucial role of strontium in the formation of a new bone matrix. It should be noted that AzR dye does not exclusively stain calcium ions since AzR can bind to other divalent cations that constitute or are present in the extracellular matrix, as in our case, enabling the determination of the global matrix mineralization degree and not just the presence of calcium deposits [39].

Hence, taking into account our results on the quantification of ALP/DNA activity, it can be suggested that PA regulates osteogenic functions that favour bone-forming processes (Figure 7). For its part, metallic phytates may allow for complementary control of osteogenic properties after 14 days. ZnPhy enabled the maintenance of normal cellular function for tissue mineralization at 21 days and remarkably increased the ALP activity at earlier times (7 and 14 days), while SrPhy significantly promoted the deposition of a homogeneous calcified matrix after 21 days (Figure 8). Therefore, our research showed the potential of PA for its application in bone pathologies affected by oxidative environments and its capacity to form bioactive phytate complexes with tailored osteogenic and antioxidant properties.

## 4. Conclusions

The results obtained in this study contribute to the further understanding of the role of PA and PA complexes (SrPhy and ZnPhy) in hMSC differentiation into an osteoblast phenotype for its potential application as cell-signalling factors in bone tissue engineering. We demonstrated the versatility of the bioactive metallic complexes that showed a complementary effect between phytate rings and M^2+^ cations in the regulation of antioxidant and osteogenic properties of PA *in vitro*. Both complex derivatives exhibited ion exchange ability in the presence of iron ions, as determined by the ferrozine/FeCl_2_ system with a dose-increasing trend, while only ZnPhy was able to reduce the content of free radicals in the DPPH assay. RAW264.7 cultures revealed that all compounds inhibited lipid peroxidation following the decreasing order PA > SrPhy > ZnPhy. ZnPhy not only hindered lipid peroxidation but also reduced ROS production by 20% using activated macrophages. All the phytate compounds were not cytotoxic in the range of 2.5–30 µg/mL when tested *in vitro* with each of the hMSCs and RAW264.7 cultures; furthermore, particular doses of phytate complexes increased the cytocompatibility in comparison with precursor PA. The cell viability of hMSCs was not compromised by the phytate compounds at 5 and 10 µg/mL after 21 days of culture, and even a significant enhancement was observed for ZnPhy and PA. Furthermore, the phytate compounds were able to modulate the osteogenic differentiation of hMSCs into an osteoblastic phenotype in terms of ALP activity levels and the matrix mineralization degree. In particular, 5 and 10 µg/mL doses of ZnPhy overexpressed ALP activity at 3 and 14 days, and SrPhy significantly improved the matrix deposition at all study times at 10 µg/mL after 21 days. For its part, PA increased both the ALP activity and matrix mineralization, exhibiting an increasing effect with the concentration of PA. Finally, it could be concluded that PA and the metallic phytate complexes exhibited appropriate *in vitro* properties for the regeneration of bone tissues in oxidative environments, making them suitable as osteoinductive factors for biomedical applications.

## Data Availability

Raw data are available upon request from the corresponding author.

## Abbreviations

ALP	Alkaline phosphatase
AzR	Alizarin red
basal-DMEM	Basal medium Dulbecco’s Modified Eagle’s Medium
BMD	Bone mineral density
CPC	Cetylpyridinium chloride
DCF	Dichlorofluorescein
DCFH-DA	2,7′-Dichlorodihydrofluorescein diacetate
DMEM-MHG	Dulbecco’s Modified Eagle’s Medium-high glucose
DPPH	2,2-diphenyl-1-picrylhydrazyl
hMSC	Human mesenchymal stem cells
EDTA	Ethylenediaminetetraacetic acid
FBS	Fetal bovine serum
hUC-MSCs	Human umbilical cord mesenchymal stem cells
MDA	Malondialdehyde
MSCM	Mesenchymal Stem Cell Medium Kit
OPG	Osteoprotegerin, osteogenic-DMEM-LG, osteogenic medium DMEM Medium-low glucose
PA	Phytic acid
PBS	Phosphate-buffered saline
ROS	Reactive oxygen species
RANK	Receptor activator of nuclear factor kappa Β
RANKL	Receptor activator of nuclear factor kappa Β ligand
RAW264.7	Murine macrophage cell line
RSA	Radical scavenger activity
SrPhy	Strontium phytate
SD	Standard deviation
TNF	Tumour necrosis factor
ZnPhy	Zinc phytate

## References

[1] Liu Z.-Q. (2020). Bridging free radical chemistry with drug discovery: A promising way for finding novel drugs efficiently. Eur. J. Med. Chem..

[2] Choukroun E., Surmenian J., Simonpieri A., Choukroun J. (2011). Oxidative Stress and Osteoimmunology: The two Missing Pieces of the Oral Osseointegration Puzzle. Immunol. Res. Ther. J..

[3] Domazetovic V., Marcucci G., Iantomasi T., Brandi M.L., Vincenzini M.T. (2017). Oxidative stress in bone remodeling: Role of antioxidants. Clin. Cases Miner. Bone Metab..

[4] Baek K.H., Oh K.W., Lee W.Y., Lee S.S., Kim M.K., Kwon H.S., Rhee E.J., Han J.H., Song K.H., Cha B.Y. (2010). Association of oxidative stress with postmenopausal osteoporosis and the effects of hydrogen peroxide on osteoclast formation in human bone marrow cell cultures. Calcif. Tissue Int..

[5] Tian Y., Ma X., Yang C., Su P., Yin C., Qian A.-R. (2017). The impact of oxidative stress on the bone system in response to the space special environment. Int. J. Mol. Sci..

[6] Yasuda H. (2021). Discovery of the RANKL/RANK/OPG system. J. Bone Miner. Metab..

[7] Castaneda O.A., Lee S.-C., Ho C.-T., Huang T.-C. (2017). Macrophages in oxidative stress and models to evaluate the antioxidant function of dietary natural compounds. J. Food Drug Anal..

[8] Boyce B.F., Xing L. (2008). Functions of RANKL/RANK/OPG in bone modeling and remodeling. Arch. Biochem. Biophys..

[9] Li H., Qi Z., Zheng S., Chang Y., Kong W., Fu C., Yu Z., Yang X., Pan S. (2019). The Application of Hyaluronic Acid-Based Hydrogels in Bone and Cartilage Tissue Engineering. Adv. Mater. Sci. Eng..

[10] Catalá A. (2009). Lipid peroxidation of membrane phospholipids generates hydroxy-alkenals and oxidized phospholipids active in physiological and/or pathological conditions. Chem. Phys. Lipids..

[11] Ayala A., Muñoz M., Argüelles S. (2014). Lipid peroxidation: Production, metabolism, and signaling mechanisms of malondialdehyde and 4-hydroxy-2-nonenal. Oxidative Med. Cell. Longev..

[12] Sasaki C.J., Cottam G.L. (1983). Lipid peroxidation following thermal injury. J. Burn Care Rehabil..

[13] Gaschler M.M., Stockwell B.R. (2017). Lipid peroxidation in cell death. Biochem. Biophys. Res. Commun..

[14] Liu L., Liang X.-F., Li J., Yuan X., Fang J. (2017). Effects of supplemental phytic acid on the apparent digestibility and utilization of dietary amino acids and minerals in juvenile grass carp ( Ctenopharyngodon idellus ). Aquac. Nutr..

[15] Qin J., Shi X., Li H., Zhao R., Li G., Zhang S., Ding L., Cui X., Zhao Y., Zhang R. (2022). Performance and failure process of green recycling solutions for preparing high degradation resistance coating on biomedical magnesium alloys. Green Chem..

[16] Erdman J.W., Poneros-Schneier A. (1989). Phytic acid interactions with divalent cations in foods and in the gastrointestinal tract. Adv. Exp. Med. Biol..

[17] Serraino M.R., Thompson L.U. (1984). Removal of Phytic Acid and Protein-Phytic Acid Interactions in Rapeseed. J. Agric. Food Chem..

[18] Zajdel A., Wilczok A., Wȩglarz L., Dzierżewicz Z. (2013). Phytic acid inhibits lipid peroxidation in vitro. Biomed Res. Int..

[19] Graf E., Empson K.L., Eaton J.W. (1987). Phytic acid. A natural antioxidant. J. Biol. Chem..

[20] Ko K.M., Godin D.V. (1990). Ferric ion-induced lipid peroxidation in erythrocyte membranes: Effects of phytic acid and butylated hydroxytoluene. Mol. Cell. Biochem..

[21] Lee B.J., Hendricks D.G. (1995). Phytic Acid Protective Effect Against Beef Round Muscle Lipid Peroxidation. J. Food Sci..

[22] del Mar Arriero M., Ramis J.M., Perelló J., Monjo M. (2012). Inositol hexakisphosphate inhibits osteoclastogenesis on RAW 264.7 cells and human primary osteoclasts. PLoS ONE.

[23] Del Mar Arriero M., Ramis J.M., Perelló J., Monjo M. (2012). Differential response of MC3T3-E1 and human mesenchymal stem cells to inositol hexakisphosphate. Cell. Physiol. Biochem..

[24] Addison W.N., McKee M.D. (2010). Inositol hexakisphosphate inhibits mineralization of MC3T3-E1 osteoblast cultures. Bone.

[25] Zhang H., Liu K., Lu M., Liu L., Yan Y., Chu Z., Ge Y., Wang T., Qiu J., Bu S. (2021). Micro/nanostructured calcium phytate coating on titanium fabricated by chemical conversion deposition for biomedical application. Mater. Sci. Eng. C.

[26] Mora-Boza A., López-Donaire M.L., Saldaña L., Vilaboa N., Vázquez-Lasa B., Román J.S. (2019). Glycerylphytate compounds with tunable ion affinity and osteogenic properties. Sci. Rep..

[27] Mora-Boza A., García-Fernández L., Barbosa F.A., Oliveira A.L., Vázquez-Lasa B., Román J.S. (2020). Glycerylphytate crosslinker as a potential osteoinductor of chitosan-based systems for guided bone regeneration. Carbohydr. Polym..

[28] Lopez-Gonzalez A.A., Grases F., Perello J., Tur F., Costa-Bauza A., Monroy N., Mari B., Vicente-Herrero T. (2010). Phytate levels and bone parameters: A retrospective pilot clinical trial. Front. Biosci..

[29] Grases F., Sanchis P., Prieto R.M., Perelló J., López-González Á.A. (2010). Effect of Tetracalcium Dimagnesium Phytate on Bone Characteristics in Ovariectomized Rats. J. Med. Food..

[30] Yang F., Yang D., Tu J., Zheng Q., Cai L., Wang L. (2011). Strontium enhances osteogenic differentiation of mesenchymal stem cells and in vivo bone formation by activating Wnt/catenin signaling. Stem Cells.

[31] Peng S., Zhou G., Luk K.D.K., Cheung K., Li Z., Lam W.M., Zhou Z., Lu W.W. (2009). Strontium promotes osteogenic differentiation of mesenchymal stem cells through the Ras/MAPK signaling pathway. Cell Physiol. Biochem..

[32] Jiménez M., Abradelo C., Román J.S., Rojo L. (2019). Bibliographic review on the state of the art of strontium and zinc based regenerative therapies. Recent developments and clinical applications. J. Mater. Chem. B.

[33] Baltaci A.K., Yuce K., Mogulkoc R. (2018). Zinc Metabolism and Metallothioneins. Biol. Trace Elem. Res..

[34] Cabrera W.E., Schrooten I., De Broe M.E., D’Haese P.C. (1999). Strontium and Bone. J. Bone Miner. Res..

[35] Marie P.J. (2006). Strontium ranelate: A physiological approach for optimizing bone formation and resorption. Bone.

[36] Rojo L., Radley-Searle S., Fernandez-Gutierrez M., Rodriguez-Lorenzo L.M., Abradelo C., Deb S., Roman J.S. (2015). The synthesis and characterisation of strontium and calcium folates with potential osteogenic activity. J. Mater. Chem. B.

[37] Powell S.R. (2000). The Antioxidant Properties of Zinc. J. Nutr..

[38] Seo H.-J., Cho Y.-E., Kim T., Shin H.-I., Kwun I.-S. (2010). Zinc may increase bone formation through stimulating cell proliferation, alkaline phosphatase activity and collagen synthesis in osteoblastic MC3T3-E1 cells. Nutr. Res. Pract..

[39] Fernández-Villa D., Asensio G., Silva M., Ramírez-Jiménez R.A., Saldaña L., Vilaboa N., Leite-Oliveira A., San Román J., Vázquez-Lasa B., Rojo L. (2021). Vitamin B9 derivatives as carriers of bioactive cations for musculoskeletal regeneration applications: Synthesis, characterization and biological evaluation. Eur. J. Med. Chem..

[40] Martín-Del-Campo M., Sampedro J.G., Flores-Cedillo M.L., Rosales-Ibañez R., Rojo L. (2019). Bone regeneration induced by strontium folate loaded biohybrid scaffolds. Molecules.

[41] Martin-del-campo M., Rosales-ibañez R., Alvarado K., Sampedro J.G., Garcia-Sepulveda C.A., Deb S., San Román J., Rojo L. (2016). Strontium folate loaded biohybrid scaffolds seeded with dental pulp stem cells induce in vivo bone regeneration in critical sized defects. Biomater. Sci..

[42] Asensio G., Benito-Garzón L., Ramírez-Jiménez R.A., Guadilla Y., Gonzalez-Rubio J., Abradelo C., Parra J., Martín-López M.R., Aguilar M.R., Vázquez-Lasa B. (2022). Biomimetic Gradient Scaffolds Containing Hyaluronic Acid and Sr/Zn Folates for Osteochondral Tissue Engineering. Polymers.

[43] Asensio G., Hernández-Arriaga A.M., Martín-Del-Campo M., Prieto M.A., Rojo L., Vázquez-Lasa B. (2022). A study on Sr/Zn phytate complexes: Structural properties and antimicrobial synergistic effects against Streptococcus mutans. Sci. Rep..

[44] Dinis T.C., Maderia V.M., Almeida L.M. (1994). Action of Phenolic Derivatives (Acetaminophen). Arch. Biochem. Biophys..

[45] Carragee E.J., Hurwitz E.L., Weiner B.K. (2011). A critical review of recombinant human bone morphogenetic protein-2 trials in spinal surgery: Emerging safety concerns and lessons learned. Spine J..

[46] Cooper C., Fox K.M., Borer J.S. (2014). Ischaemic cardiac events and use of strontium ranelate in postmenopausal osteoporosis: A nested case-control study in the CPRD. Osteoporos. Int..

[47] Abrahamsen B., Grove E., Vestergaard P. (2014). Nationwide registry-based analysis of cardiovascular risk factors and adverse outcomes in patients treated with strontium ranelate. Osteoporos. Int..

[48] Milkovic L., Gasparovic A.C., Cindric M., Mouthuy P.-A., Zarkovic N. (2019). Short Overview of ROS as Cell Function Regulators and Their Implications in Therapy Concepts. Cells.

[49] Bagchi D., Bagchi M., Stohs S. (1997). Comparative in vitro oxygen radical scavenging ability of zinc methionine and selected zinc salts and antioxidants. Gen. Pharmacol..

[50] Prasad A.S. (2014). Zinc is an Antioxidant and Anti-Inflammatory Agent: Its Role in Human Health. Front. Nutr..

[51] Halliwell B., Chirico S. (1993). Lipid peroxidation: Significance and its mechanism. Am. J. Clin. Nutr..

[52] Toyokuni S. (2002). Iron and carcinogenesis: From Fenton reaction to target genes. Redox Rep..

[53] Torres J., Domínguez S., Cerdá M.F., Obal G., Mederos A., Irvine R.F., Díaz A., Kremer C. (2005). Solution behaviour of myo -inositol hexakisphosphate in the presence of multivalent cations. Prediction of a neutral pentamagnesium species under cytosolic/nuclear conditions. J. Inorg. Biochem..

[54] Marolt G., Gričar E., Pihlar B., Kolar M. (2020). Complex Formation of Phytic Acid With Selected Monovalent and Divalent Metals. Front. Chem..

[55] Kim H., Lee Y.D., Kim H.J., Lee Z.H., Kim H.-H. (2017). SOD2 and Sirt3 Control Osteoclastogenesis by Regulating Mitochondrial ROS. J. Bone Miner. Res..

[56] Callaway D.A., Jiang J.X. (2015). Reactive oxygen species and oxidative stress in osteoclastogenesis, skeletal aging and bone diseases. J. Bone Miner. Metab..

[57] El-Refai A.A., Ghoniem G.A., El-Khateeb A.Y., Hassaan M.M. (2018). Eco-friendly synthesis of metal nanoparticles using ginger and garlic extracts as biocompatible novel antioxidant and antimicrobial agents. J. Nanostructure Chem..

[58] Elkodous M.A., El-Sayyad G.S., Maksoud M.I.A.A., Abdelrahman I.Y., Mosallam F.M., Gobara M., El-Batal A.I. (2020). Fabrication of Ultra-Pure Anisotropic Zinc Oxide Nanoparticles via Simple and Cost-Effective Route: Implications for UTI and EAC Medications. Biol. Trace Elem. Res..

[59] Rajeshkumar S., Kumar S.V., Ramaiah A., Agarwal H., Lakshmi T., Roopan S.M. (2018). Biosynthesis of zinc oxide nanoparticles usingMangifera indica leaves and evaluation of their antioxidant and cytotoxic properties in lung cancer (A549) cells. Enzym. Microb. Technol..

[60] Press D. (2015). Novel conductive polypyrrole / zinc oxide/chitosan bionanocomposite: Synthesis, characterization, antioxidant, and antibacterial activities. Int. J. Nanomedicine..

[61] Wu S., Du Y., Hu Y., Shi X., Zhang L. (2013). Antioxidant and antimicrobial activity of xylan-chitooligomer-zinc complex. Food Chem..

[62] Wu C., Zheng X.P., Chen L.L. (2011). Study on antioxidant activity of dihydromyricetin-zinc(II) complex. Adv. Mater. Res..

[63] Baran A., Karakılıç E., Faiz Ö., Özen F. (2020). Synthesis of chalcone-containing zinc and cobalt metallophthalocyanines; investigation of their photochemical, DPPH radical scavenging and metal chelating characters. Org. Commun..

[64] Fuhrman B., Oiknine J., Aviram M. (1994). Iron induces lipid peroxidation in cultured macrophages, increases their ability to oxidatively modify LDL, and affects their secretory properties. Atherosclerosis.

[65] Tsikas D. (2017). Assessment of lipid peroxidation by measuring malondialdehyde (MDA) and relatives in biological samples: Analytical and biological challenges. Anal. Biochem..

[66] Moore K., Roberts L.J. (1998). Measurement of lipid peroxidation. Free Radic. Res..

[67] Huntosova V., Horvath D., Seliga R., Wagnieres G. (2021). Influence of oxidative stress on time-resolved oxygen detection by [ru(Phen)3 ]2+ in vivo and in vitro. Molecules.

[68] Bose C., Hindle A., Lee J., Kopel J., Tonk S., Palade P.T., Singhal S.S., Awasthi S., Singh S.P. (2021). Anticancer activity of Ω-6 fatty acids through increased 4-hne in breast cancer cells. Cancers.

[69] Del Favero G., Hohenbichler J., Mayer R.M., Rychlik M., Marko D. (2020). Mycotoxin Altertoxin II Induces Lipid Peroxidation Connecting Mitochondrial Stress Response to NF-κB Inhibition in THP-1 Macrophages. Chem. Res. Toxicol..

[70] Sullivan J.F., Jetton M.M., Hahn H.K., Burch R.E. (1980). Enhanced lipid peroxidation in liver microsomes of zinc-deficient rats. Am J Clin Nutr..

[71] Tunçdemir M., Ertürküner S.P., Özçelik D. (2017). Investigation of lipid peroxidation and antiapoptotic effects of zinc aganist liver damage in diabetic rats. Hum. Exp. Toxicol..

[72] Zago M.P., Verstraeten S.V., Oteiza P.I. (2000). Zinc in the prevention of Fe2+-initiated lipid and protein oxidation. Biol. Res..

[73] Irvine R.F., Schell M.J. (2001). Back in the water: The return of the inositol phosphate. Nat. Rev. Mol. Cell Biol..

[74] Liu Y., Wu J., Zhang H., Wu Y., Tang C. (2021). Covalent immobilization of the phytic acid-magnesium layer on titanium improves the osteogenic and antibacterial properties. Colloids Surfaces B Biointerfaces..

[75] Wang Y., Lou J., Zeng L., Xiang J., Zhang S., Wang J., Xiong F., Li C., Zhao Y., Zhang R. (2017). Osteogenic potential of a novel microarc oxidized coating formed on Ti6Al4V alloys. Appl. Surf. Sci..

[76] Wang Q., Ding C., Zhou Y., Luo J., Li J. (2018). Universal and biocompatible hydroxyapatite coating induced by phytic acid-metal complex multilayer. Colloids Surf. B Biointerfaces.

[77] Li J., Wang S., Dong Y. (2021). Regeneration of pulp-dentine complex-like tissue in a rat experimental model under an inflammatory microenvironment using high phosphorous-containing bioactive glasses. Int. Endod. J..

[78] Bonnelye E., Chabadel A., Saltel F., Jurdic P. (2008). Dual effect of strontium ranelate: Stimulation of osteoblast differentiation and inhibition of osteoclast formation and resorption in vitro. Bone.

[79] Capuccini C., Torricelli P., Sima F., Boanini E., Ristoscu C., Bracci B., Socol G., Fini M., Mihailescu I., Bigi A. (2008). Strontium-substituted hydroxyapatite coatings synthesized by pulsed-laser deposition: In vitro osteoblast and osteoclast response. Acta Biomater..

[80] Park J.-W., Kim H.-K., Kim Y.-J., Jang J.-H., Song H., Hanawa T. (2010). Osteoblast response and osseointegration of a Ti-6Al-4V alloy implant incorporating strontium. Acta Biomater..

